# Genetic Analysis of Soybean Flower Size Phenotypes Based on Computer Vision and Genome-Wide Association Studies

**DOI:** 10.3390/ijms25147622

**Published:** 2024-07-11

**Authors:** Song Jin, Huilin Tian, Ming Ti, Jia Song, Zhenbang Hu, Zhanguo Zhang, Dawei Xin, Qingshan Chen, Rongsheng Zhu

**Affiliations:** 1 College of Agriculture, Northeast Agricultural University, Harbin 150030, Chinadwxin@neau.edu.cn (D.X.); 2 National Key Laboratory of Smart Farm Technolog and System, Harbin 150030, China; 3 College of Arts and Sciences, Northeast Agricultural University, Harbin 150030, China

**Keywords:** phenomics, computer vision, genome-wide association studies, haplotype analysis, soybean organ development

## Abstract

The dimensions of organs such as flowers, leaves, and seeds are governed by processes of cellular proliferation and expansion. In soybeans, the dimensions of these organs exhibit a strong correlation with crop yield, quality, and other phenotypic traits. Nevertheless, there exists a scarcity of research concerning the regulatory genes influencing flower size, particularly within the soybean species. In this study, 309 samples of 3 soybean types (123 cultivar, 90 landrace, and 96 wild) were re-sequenced. The microscopic phenotype of soybean flower organs was photographed using a three-eye microscope, and the phenotypic data were extracted by means of computer vision. Pearson correlation analysis was employed to assess the relationship between petal and seed phenotypes, revealing a strong correlation between the sizes of these two organs. Through GWASs, SNP loci significantly associated with flower organ size were identified. Subsequently, haplotype analysis was conducted to screen for upstream and downstream genes of these loci, thereby identifying potential candidate genes. In total, 77 significant SNPs associated with vexil petals, 562 significant SNPs associated with wing petals, and 34 significant SNPs associated with keel petals were found. Candidate genes were screened by candidate sites, and haplotype analysis was performed on the candidate genes. Finally, the present investigation yielded 25 and 10 genes of notable significance through haplotype analysis in the vexil and wing regions, respectively. Notably, *Glyma.07G234200*, previously documented for its high expression across various plant organs, including flowers, pods, leaves, roots, and seeds, was among these identified genes. The research contributes novel insights to soybean breeding endeavors, particularly in the exploration of genes governing organ development, the selection of field materials, and the enhancement of crop yield. It played a role in the process of material selection during the growth period and further accelerated the process of soybean breeding material selection.

## 1. Introduction

Soybeans [*Glycine max* (L.) Merr.] are widely cultivated all over the world and are a vital source of plant protein, oil, and food [1,2,3]. Cultivating stable high-yield and high-quality soybeans has consistently been a significant research objective of breeding scientists. The size of organs in plants has a considerable impact on yield and quality [4]. In soybean anatomy, seeds serve as a crucial food source for both humans and animals. Delving into the intricate mechanisms underpinning soybean organ development and augmenting soybean yield stands as a pivotal approach to addressing the food scarcity challenges stemming from population expansion.

The growth and development of plant organs are controlled by inheritance [5]. The size of the organ is primarily affected by the growth of two basic cellular processes, namely, cell proliferation and cell expansion [6]. In research on model species, certain genes regulating organ growth have been identified [7,8]. The flower stands as a pivotal reproductive structure in plants. Petal size and morphology wield significant influence over pollination dynamics and exert considerable impact on overall plant growth and development [9]. The dimensions of the flower are likely influenced by the coordinated regulation of genes associated with organ size across the plant [10]. Nonetheless, in the field of soybean research, investigations into genes regulating soybean flower size are relatively sparse. This scarcity is partly due to the microscopic nature of soybean flower phenotypes, which complicates their extraction and necessitates substantial time investments, thus limiting the extent of studies in this area [11]. There is a lack of research on genes related to the excavation of soybean petal traits. With the development of computer vision technology, there are new methods to obtain phenotypes that cannot be measured in the past [12]. For example, they have applications in many fields of plant phenotype, such as Populus trichocarpa, Arabidopsis, etc., and rice, maize, and wheat crops. A number of published studies have used various computer vision methods in plant GWASs [13,14,15].

In 2000, Yukiko Mizukami and Robert L. Fischer et al. found that the *Arabidopsis* regulatory gene *AINTEGUMENATA (ANT)* controls the number of plant organ cells and organ size during bud development [16]. In 2011, Guanping Feng et al. observed significant enlargement in the leaves, cotyledons, and flower organs of transgenic *35S-OSR1* plants in *Arabidopsis thaliana* [7]. In 2012, Shengjun Li et al. discovered that *35S:AGG3* transgenic plants exhibited elongated grains, increased seed quantity, and had a larger seed size compared to wild-type plants [8]. In addition to the research in *Arabidopsis*, previous researchers have also found this phenomenon in legumes. In 2021, Shaoli Zhou and Tianquan Yang et al. identified that the *MIO1/SLB1* gene could modulate the size of leaves, flowers, seeds, and other organs in *Medicago truncatula* [17]. However, in soybeans, petals are a microscopic phenotype, and there are few related analytical reports.

The genomes of many crop populations have been re-sequenced using next-generation sequencing (NGS) techniques. The utilization of whole-genome re-sequencing data is deemed essential for conducting Genome-Wide Association Studies (GWASs), a method widely employed in the investigation of various crop species [18,19]. Compared with linkage maps, a GWAS has the advantages of high-density resolution and is suitable for a large number of markers and population size analysis. In addition, a GWAS also uses better statistical methods to analyze association mapping between the traits of interest and the number of single nucleotide polymorphism (SNP) markers based on the resource population and linkage disequilibrium (LD) structure [20,21]. A GWAS has been extensively adopted for the analysis of multiple traits of major grain crops, such as maize and rice [22,23]. In soybeans, numerous traditional traits such as Days to Flowering, Maturity, Flowering-to-Maturity Duration, Plant Height, Seed Weight, Protein Content, Oil Content, Disease Resistance, Flood Tolerance, and Salt Tolerance have been mapped to SNPs or quantitative trait loci (QTL) associated with these traits [24,25,26,27,28,29]. Through the screening of genes within the vicinity of significant SNP loci, genes related to multiple complex traits have been identified [30]. This has significantly accelerated the advancement of soybean breeding efforts.

In order to explore the SNP loci related to the trait of soybean petals and screen the related genes through SNP loci, we re-sequenced 309 samples in the soybean resource population using NGS technology to obtain SNP data. The microscopic phenotype of soybean petals was photographed using a microscope, and the phenotype had a high throughput and was extracted by computer vision technology. A GWAS was performed on soybean petal-related phenotypic data and resource population SNP data. The SNP loci and genes related to the high size of soybean petals were explored through the aforementioned methods. We analyzed the relationship between soybean flowers and seeds and demonstrated the correlation between them with data and results from both phenotypic and genetic perspectives. We believe that this can play a huge role in early material selection in soybean breeding and further accelerate the process of material selection.

## 2. Results

### 2.1. Phenotypic Correlation Analysis

**Figure 1:**
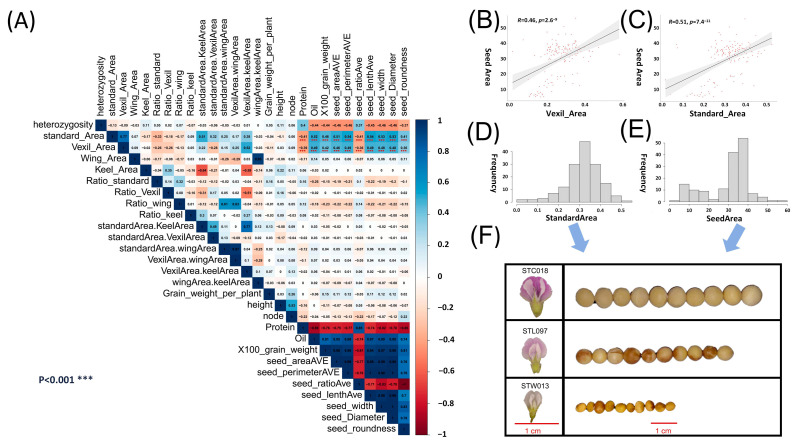
Correlation analysis and analysis of the petal phenotype and seed phenotype. A petal trait GWAS. (**A**) Pearson correlation analysis of the petal phenotype and seed phenotype in natural populations; red represents a positive correlation, blue represents a negative correlation; *** denotes a correlation *p* value < 0.001. (**B**) Scatter plot of the vexil area and seed area correlation analysis. (**C**) Scatter plot of the standard area and seed area correlation analysis. (**D**) Standard area phenotypic data frequency distribution histogram. (**E**) Seed area phenotypic data frequency distribution histogram. (**F**) Samples were selected according to the size of the seed area and standard area phenotype, and the corresponding relationship between grains and petals was observed. The red line represents the 1 cm reference line.

Pearson correlation analysis was performed on the obtained petal phenotypic data (Supplementary Table S1) and seed phenotypic data (Supplementary Table S2) (Figure 1A). The results show that the 100-grain weight, seed area, seed perimeter, seed length, and seed width in the seed phenotype were related to the standard area (SA) and vexil area (VA) in the petal phenotype. The correlation r values were 0.40–0.54, and the *p* values were less than 0.001 (Figure 1A). Using canonical correlation analysis, the first correlation coefficient of 0.5969 was obtained for the aforementioned five seed phenotypes and four types of petal area phenotypes with high correlation. In Figure 1C, the correlation analysis results between the seed area and SA reveal a significant positive correlation (r = 0.51, *p* value = 7.4 × 10^−11^). Subsequently, histograms were constructed for both traits (Figure 1D,E), with representative samples taken for large, medium, and small categories in each phenotype. Specifically, sample STC018, characterized by large grains and petals, fell within the range of 0.4–0.45 for the SA and 40–45 for the seed area. Sample STL097, representing medium grains and petals, was situated in the range of 0.3–0.35 for the SA and 30–35 for the seed area. Lastly, sample STW013, denoting small grains and petals, fell within the range of 0.15–0.20 for the SA and 10–15 for the seed area. Comparing these samples revealed a consistent pattern, indicating a correspondence between organ sizes, wherein samples with larger petals exhibited larger seeds (Figure 1F). Pairwise statistics were conducted on the petal and seed phenotypes based on their average values, revealing that samples with larger petal areas were more likely to correspond to samples with larger seed areas. Specifically, in the data analysis involving the vexil petal area and seed perimeter, it was observed that there were 63 instances where large-area vexil petal samples corresponded to large-perimeter seed samples. In contrast, only 11 instances were found where large-area vexil petal samples corresponded to small-perimeter seed samples. This finding further supports the conclusion that larger flower organs in plants tend to correspond to larger seed organs (Supplementary Table S3).

### 2.2. Petal and Seed Trait GWASs

**Figure 2:**
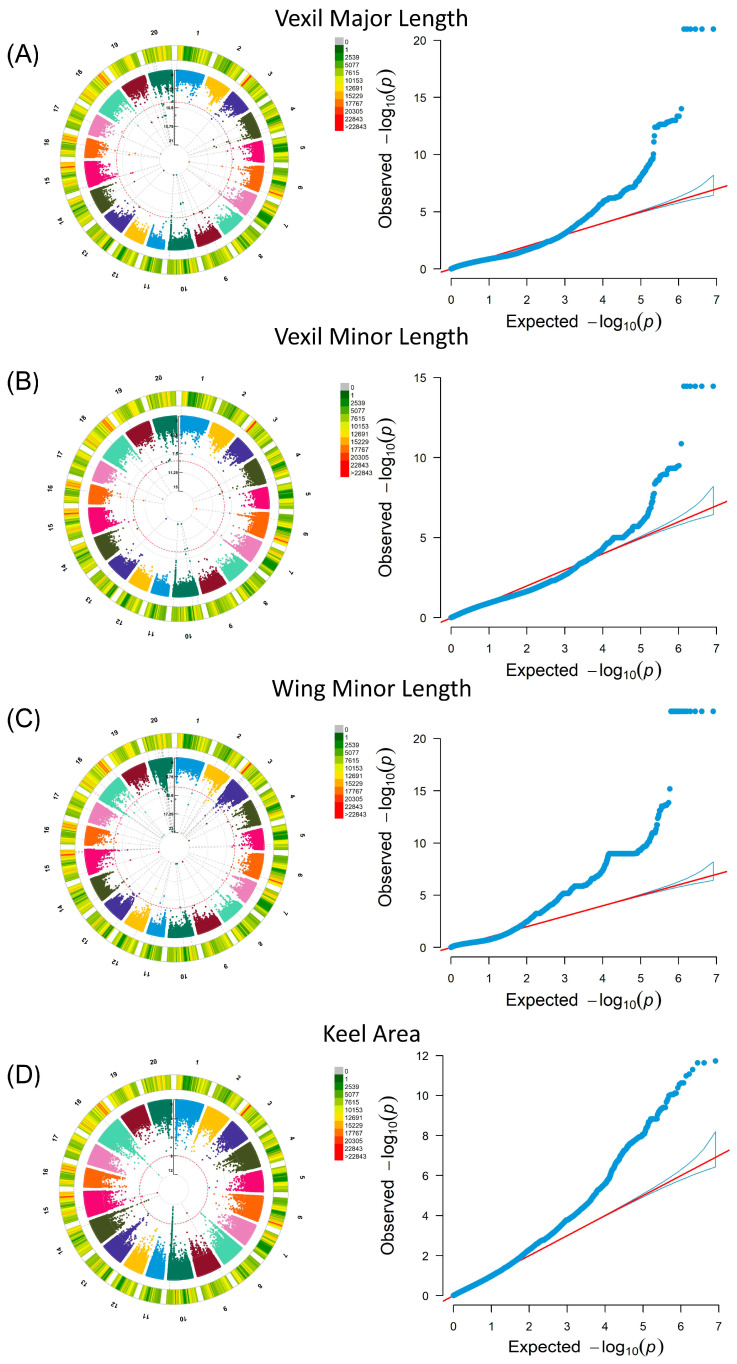
Genome-wide association analysis of petal phenotypes in the soybean natural population, featuring Circular Manhattan plots and QQ plots. In each Manhattan plot, the red line signifies the threshold. (**A**) Vexil major length was analyzed using the MLM model. (**B**) Vexil minor length was also analyzed using the MLM model. (**C**) Wing minor length underwent analysis through the MLM model. (**D**) The keel area was analyzed using the MLM model.

A GWAS was performed in a linear mixed model MLM using petal and seed phenotypic data, which involved population structure and family correlation (K matrix). The results of the seed phenotype are shown in Supplementary Figure S1. In the GWAS results of 24 petal phenotypes (Supplementary Figure S2), the selection criteria focused on SNP aggregations with site *p* values below the threshold (0.01 divided by the number of independent SNPs) [31]. For vexil major length (VMAL), fifty-five significant loci were identified across sixteen chromosomes, with five loci clustered on chromosome 7 (minimum *p* value: 1.03 × 10^−13^) (Figure 2A). Similarly, for vexil minor length (VMIL), twenty-two significant loci were detected across ten chromosomes, including two loci clustered on Chr07 (minimum *p* value: 4.74 × 10^−10^) (Figure 2B). Notably, two sites on Chr07 in VMIL were also observed in vexil major length, suggesting a potential impact on the flag leaf size in soybean flowers. In the case of wing minor length (WMIL), 562 significant loci were distributed across 19 chromosomes, with 25 loci clustered on chromosome 18 (minimum *p* value: 2.63 × 10^−23^) (Figure 2C). Lastly, for the keel area (KA), thirty-four significant loci were distributed across five chromosomes, with twenty-three loci clustered on chromosome 7 (minimum *p* value: 2.35 × 10^−12^) (Figure 2D).

### 2.3. Interaction Analysis of Significant SNPs between Soybean Seeds and Petal GWAS Results

**Figure 3:**
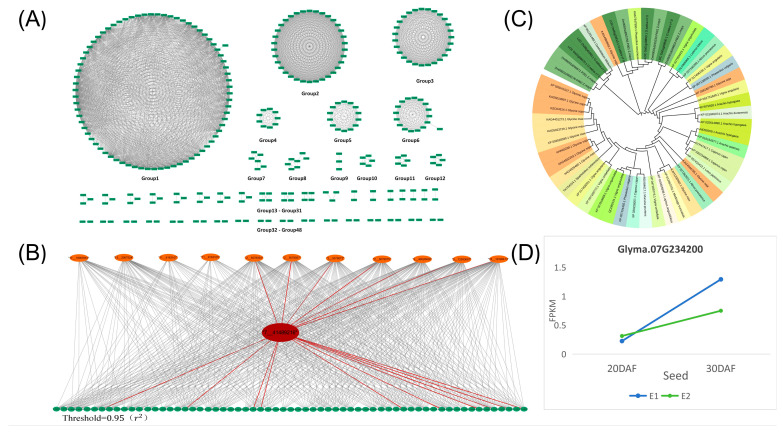
Interaction analysis of SNPs between the GWAS results of the petal phenotype and the GWAS analysis results of the seed phenotype. (**A**) The interaction diagram depicts SNPs between the petal phenotype GWAS results and the seed phenotype GWAS analysis results. Each green rectangle represents one SNP, and the gray lines denote interactions between the SNPs. The threshold was set at 0.95 (r^2^). (**B**) Interaction between the SNP locus (red ellipse) of the candidate gene *Glyma07G234200* and SNPs (orange ellipse) from the petal phenotype GWAS results and SNPs (green ellipse) from the seed phenotype GWAS results. The red lines indicate SNP loci interacting with the SNP of the candidate gene. (**C**) Phylogenetic tree analysis of the candidate gene *Glyma07G234200* with other legumes and major crops. (**D**) RNA-seq data expression analysis of large cultivar seeds (E1) and small wild seeds (E2); the seed transcriptome data of two varieties at 20 DAF and 30 DAF were sequenced, represented by the blue and green lines, respectively.

The interaction between significant SNPs identified in both the seed and petal GWAS results was examined. The analysis (Supplementary Table S4) utilized a threshold of ≥0.95. Subsequently, an interaction network diagram was constructed using Cytoscape, illustrated in Figure 3A. The 936 SNPs were categorized into 49 interaction networks. Further, a Bipartite graph (Figure 3B) was generated for Group 1 of the candidate genes shown in Figure 3A, with the SNP locus of the candidate gene *Glyma07G234200* highlighted. Notably, this locus interacts with significant SNPs from both the seed and petal GWAS results, indicating potential shared genetic regulation of the two phenotypes. The candidate gene *Glyma07G234200* was involved in this interaction. Additionally, a phylogenetic tree analysis of candidate genes across various crop species, including *O. Sativa*, *Z. mays*, *G. soja*, and *Glycine max*, revealed homologous genes of *Glyma07G234200* in multiple crop species (Figure 3C). Moreover, RNA-seq data analysis revealed differential expression patterns of *Glyma07G234200* in large-grain sample E1 and small-grain sample E2 at various stages (Figure 3D), suggesting their involvement in promoting organ development during growth.

### 2.4. Candidate Gene Identification of Vexil and Wing Traits

The strongest correlation SNP in the peak obtained by the GWAS served as the central point, with 250 K genes upstream and downstream selected as candidate genes [32]. Notably, as the peak SNP for both VMAL and VMIL was the same SNP, they shared 43 candidate genes (Supplementary Table S5). Additionally, 117 candidate genes were identified in the wing minor length results, while 107 candidate genes were identified in the keel area results (Supplementary Table S5). Subsequently, these 267 genes were annotated in Soybase, with 86 genes associated with various GO biological processes such as cell differentiation, proliferation, tip growth, cell wall biogenesis, embryo sac development, leaf and petal morphogenesis, meristem development, and morphogenesis. Further, these genes were implicated in petal and grain development and responses to plant signal transduction and biosynthetic pathways (Supplementary Table S6). Among these, 17 genes were specifically linked to flower organ development and morphogenesis.

### 2.5. Haplotype Analysis of Candidate Genes

A high-throughput gene haplotype and phenotypic significance visualization script was written using R and Shell. Haplotype analysis of SNPs was performed on the gene and CDS sequences of 267 candidate genes. In vexil petal major and vexil petal minor, 25 genes exhibited significant gene sequence variations (Supplementary Figures S3–S6), as shown in Table 1. Among these, 17 genes displayed significant gene sequence variations in both phenotypes, as detailed in Table 1. Moreover, 14 genes showed significant phenotypic variations in both the gene sequence and CDS sequence, as outlined in Table 1. Notably, *Glyma.07G234200* was previously reported to exhibit significant differences in large-grain seeds compared to small-grain seeds during the S1 stage (at 5–7 days after fertilization). *Glyma.07G233900* was identified in the co-expression of the isoflavone biosynthetic gene cluster [33]. Under aluminum stress, *Glyma.07G231800* was found to be co-expressed with genes involved in root elongation regulation, potentially affecting cell functions such as division, metabolism, and response [34]. In terms of Gene Ontology Biological Process Descriptions, *Glyma.07G231600* was associated with development, petal development, and stamen development processes. Additionally, *Glyma.07G233900* and *Glyma.07G234000* were involved in root development.

In the analysis of wing minor length, 10 genes exhibited significant phenotypic differences in the SNP haplotype analysis of gene sequences, as indicated in Table 1. Additionally, among these genes, seven had GO annotations and seven displayed significant phenotypic differences in the CDS segments (Table 1, Supplementary Figures S7 and S8). Notably, several of these genes have been previously associated with differences in plant organ size. For instance, *Glyma.18G043700* was identified in RNA sequencing analysis of seeds at Stage S3 (seeds at 20–24 days), where it was significantly up-regulated in large seeds compared to small seeds [35]. *Glyma.18G278600* was mapped to a QTL associated with heterophylly [36]. Furthermore, *Glyma.18G043700* was involved in petal and sepal formation processes according to Gene Ontology Biological Process Descriptions, while *Glyma.18G280700* was implicated in flower development processes. These genes are likely to play crucial roles in regulating the size of flowers or multiple organs.

### 2.6. Population Genetics Analysis of Candidate Genes

**Figure 4:**
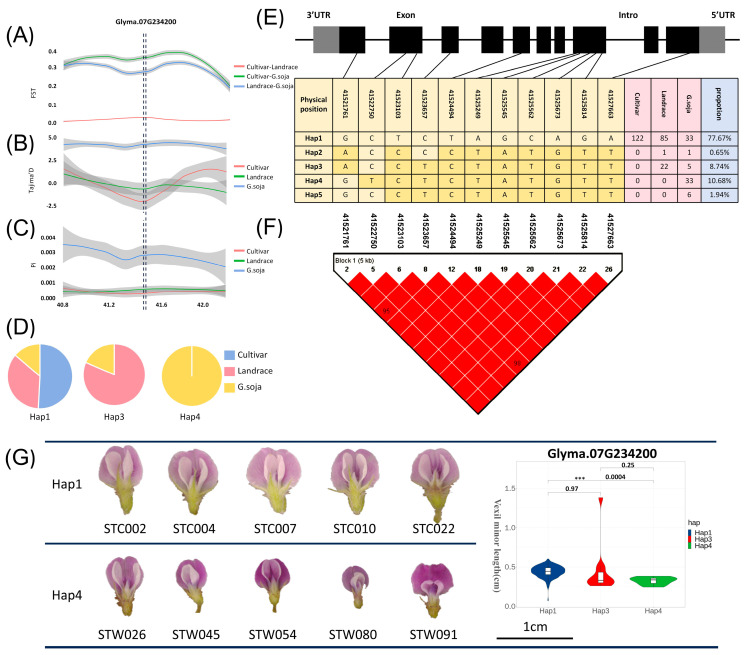
Population genetics analysis of candidate genes. (**A**) FST analysis of the candidate gene *Glyma07G234200*. Pink indicates the analysis results between cultivar and landrace populations; green represents the analysis results between cultivar and wild populations; blue represents the analysis results between wild and landrace populations. (**B**) Tajima’D analysis of the candidate gene *Glyma07G234200*. Pink indicates the results of cultivar analysis in populations; green represents the results of landrace analysis in populations; blue represents the results of wild analysis in populations. (**C**) FST analysis of the candidate gene *Glyma07G234200*. Pink represents the variety analysis results of populations; green represents the analysis results of landraces in populations; blue represents the results of wild analysis in populations. (**D**) Proportion of haplotypes in the CDS region of candidate genes in various varieties. (**E**) Gene structure of candidate genes, haplotype analysis results, and genotype of SNP loci. Gray represents the UTR region, black represents the exon region, and blank represents the intron region. The dark yellow in the table indicates the base after variation. (**F**) Linkage disequilibrium analysis of SNPs in the CDS region of candidate genes. (**G**) The haplotype analysis of the *Glyma07G234200* gene CDS sequence showed that Hap1 and Hap4 were significant in the vexil minor length phenotype. We selected samples for phenotypic verification and found that the Hap1 sample was significantly larger than the Hap4 sample. *** denotes correlation *p* value < 0.001.

The FST, Pi, and Tajima’D values in the region of *Glyma07G234200* were calculated for cultivar, landrace, and wild soybeans. In Figure 4A, the FST results aid in identifying a genetically diverse genomic region between cultivar, landrace, and wild soybeans. Genetic differentiation between wild-cultivar and wild-landrace soybeans was notably higher than that between cultivar and landrace soybeans. The Tajima’D calculation results, depicted in Figure 4B, revealed that the Tajima’D value for cultivar and landrace is negative. It indicated that there is no selection pressure within these populations. The gene may be selected during soybean improvement, resulting in its rapid accumulation in cultivar and landrace. However, the Tajima’D value for wild soybeans exceeded 0, indicating a population bottleneck or shrinkage in wild soybeans. In addition, the nucleotide diversity Pi of cultivar soybeans and landrace soybeans was also significantly smaller than that of wild soybeans, and the candidate genes may have a domestication relationship (Figure 4C). The haplotypes of the candidate gene *Glyma07G234200* were also analyzed, and it was found that among the dominant haplotypes (frequency > 5 %) [37], Hap1 contained three soybeans, Hap3 only contained wild soybeans and landrace soybeans, and Hap4 only contained wild soybeans (Figure 4D). This phenomenon further underscores the differential gene expression of *Glyma07G234200* across various categories. The gene structure of the candidate gene and the SNP location were annotated in Figure 4E. Additionally, utilizing Haploview, a linkage disequilibrium map (Figure 4F) was constructed, revealing a significant degree of linkage disequilibrium among SNPs within the CDS region. We performed haplotype analysis of the *Glyma07G234200* gene CDS sequence and found that Hap1 and Hap4 were significant in the VMIL phenotype. We selected samples for phenotypic verification and found that Hap1 was significantly larger than Hap4 (Figure 4G).

## 3. Discussion

### 3.1. Phenome and Genome Analyses Unveiled Genetic Loci Associated with Flower Organ Size in Soybeans

In the present study, distinct characteristics were observed among the resource populations of cultivar, landrace, and wild soybeans, with clearly discernible numerical distributions of phenotypic traits located by the GWAS (Supplementary Figure S9). The GWAS results (Figure 2) reveal highly correlated genetic loci within the vexil petal, wing petal, and keel petal. This research methodology’s reliability was demonstrated across various crops [23,38,39]. Despite limited investigations on genes regulating petal size, the mapped loci were found within QTL regions controlling the size of other organs. Notably, significant SNP loci for VMIL and VMAL at Chr07 41489216 fell within previously reported QTL intervals for traits such as seed length (7.84 cM–53.54 cM), seed yield (33.4 cM–50.1 cM), seed fill (38.98 cM–58.5 cM), and plant height (33.47 cM–50.09 cM) [40,41,42]. Converting identified cDNA sequence SNPs into Kompetitive Allele-Specific PCR (polymerase chain reaction) markers and integrating them into a common genetic background could offer avenues for manipulating flower petal traits in soybeans.

### 3.2. Genes That Promote Organ Development Play a Crucial Role in Regulating Soybean Yield Positively

The size of plant organs, including soybean petals, is determined by a complex mechanism involving cell proliferation and expansion [43]. Genes regulating soybean petal size may also influence other organs such as seeds, leaves, and roots through mechanisms that promote cell proliferation or expansion. In the present study, the petal area was found to be highly correlated with seed area and length (Figure 1B,C). Following GWAS localization, haplotype analysis identified numerous genes implicated in the developmental pathways of organs such as ovules, petals, and roots. Additionally, some genes associated with the regulation of transcription and cell division processes were identified. These findings suggest that the identified genes may hold significant value in soybean breeding efforts. Among the screened genes, the transcription factor *Glyma.07G234200* was pinpointed on Chr07. This gene has been previously reported as a differentially expressed gene in transcriptome analyses of grain size differences observed 5–7 days after fertilization [35]. As evidenced by ePlant data (https://bar.utoronto.ca/eplant_soybean/, accessed on 25 November 2023), *Glyma.07G234200* exhibits high expression levels across various plant organs including flowers, pods, leaves, roots, and seeds. This gene is from the SBP-box family. The SBP-box gene was first discovered in *Antirrhinum majus* because its protein product can bind to the promoter region of the floral meristem identity gene *SQUAMOSA* [44]. SBP-box genes have also been found in various plant species, and their functions have been extensively studied [45,46,47,48]. *SPL3* was highly expressed in vegetative growth and inflorescence apex, floral meristem, leaves, and floral primordia [46]. *SPL3*, *SPL4,* and *SPL5* were significantly up-regulated under long-day floral induction [49]. *SPL8* showed that it affected the development of pollen sacs [50]. SBP-box genes are ubiquitous in higher plants and play an important role in plant growth and flower development. Integrating the findings from the GWAS, haplotype analysis, previous transcriptome studies, and ePlant information, it can be inferred that the *Glyma.07G234200* gene serves as a crucial transcription factor involved in the developmental processes of various soybean organs [44]. By facilitating the expansion of soybean organs, including seeds, these genes have the potential to enhance both the yield and quality of soybean crops. The present research contributes to advancing soybean breeding efforts, offering promising avenues for further development in the field.

### 3.3. Exploration and Analysis of the General Law of Petal Size and Grain Size of Leguminous Plants

Across leguminous plants, like chickpeas, peas, and common beans, notable variations exist in the sizes of both seeds and petals. A common trend observed is that larger flowers tend to correspond to larger seeds. In the present study, the phenomenon of large flowers corresponding to large pods in legumes was preliminarily explored and analyzed, thereby providing an explanation for the study of the relationship between floral organs and the yield of legumes. The development of plant organs is affected by genetic control and the physiological activities of other organs [5]. This effect leads to a mutual promotion or mutual inhibition relationship between plant organs during the growth process, which is referred to as growth correlation [51]. However, nutrient absorption in plants is influenced by a variety of factors, categorized into internal and environmental factors [52]. Internal factors encompass attributes such as charge and ion diameter, while environmental factors encompass variables like light, temperature, water availability, oxygen levels, pH, nutrient concentrations, and the interactions among these factors [53,54,55,56,57]. In terms of the influence of the external environment, there are differences in the tolerance of each soybean variety to external environmental changes, which may lead to different degrees of change in nutrient supply, thereby leading to contrasting relationships in a select few varieties [58].

### 3.4. The Combination of Computer Vision and the GWAS Brings a New Perspective and Application for Smart Breeding

With the development of artificial intelligence, more and more people use deep learning, computer vision, and other methods to analyze the genetics of plants. In crops, researchers used the Python/OpenCV library (https://github.com/openalea/phenomenal (accessed on 4 July 2024)) to analyze images to calculate the transpiration rate of *O. glaberrima*. At least 14 potential genetic regions associated with water use-related traits in *O. glaberrima* were mapped by the GWAS [13]. Some researchers used deep learning to visually extract the phenotype of soybean sudden death syndrome disease severity and mapped 46 significant SNPs related to disease severity through the GWAS [59]. Whether it is deep learning or OpenCV, the extracted phenotypes are phenotypes that can be extracted manually, and computer vision only increases work efficiency. However, in maize, some researchers used computer vision to extract the shank and vascular bundle phenotypes that cannot be accurately extracted manually and found 806 SNPs related to them [14]. In this study, we used computer vision technology to accurately extract and analyze the soybean petal phenotype that could not be accurately extracted in the past. A total of 673 SNPs related to petal size were obtained, and the correlation between petals and seeds was analyzed phenotypically and genetically. The phenotype is the core technology of intelligent breeding. By analyzing the micro-phenotype, the selection of breeding materials can be accelerated. In summary, we proposed a computer vision and a GWAS approach to analyze the microscopic phenotype of soybean petals and realized the analysis of crop microscopic phenotypes. This method can be applied to multiple crops to provide new solutions for smart breeding.

## 4. Materials and Methods

**Figure 5:**
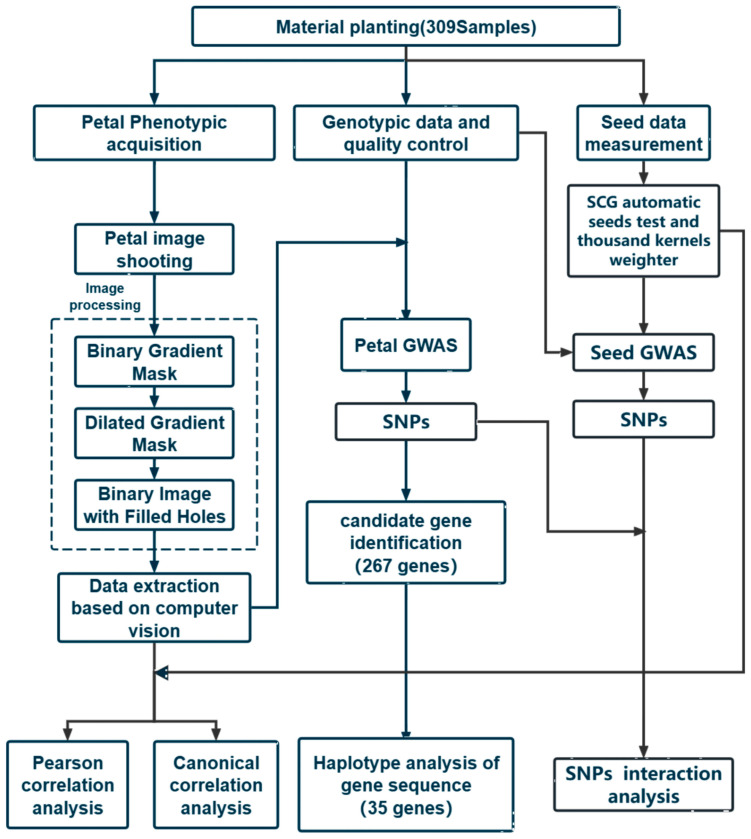
Technical route of this study.

The overall technical route and process used in the research institute are shown in Figure 5.

### 4.1. Material Planting

The materials used in the present study included 309 re-sequenced soybean varieties (Supplementary Table S7) covering 3 types: cultivar, landrace, and wild. The ratio of the three types of soybeans was 123:90:96. In the present study, all experimental materials were planted at the Xiangyang Base of Northeast Agricultural University in Harbin, Heilongjiang Province (126.9 E, 45.7 N), in 2023. The experimental planting involved a single-row configuration (each row spanning 5 m with a spacing of 0.66 m; individual seed spacing set at 10 cm). Following soybean flowering, individual plants from each variety were randomly chosen, and fully open soybean flowers were sampled for subsequent measurements.

### 4.2. Genotype Data Acquisition

Whole Genome Re-sequencing (WGRS) was performed on 309 re-sequenced soybean resources using the next-generation sequencing technology of The Beijing Genomics Institute’s BGI platform with a sequencing depth of 30× [60]. The sequencing data underwent processing via the BWA-GATK4 (version gatk-4.1.3.0) pipeline [61], utilizing the *Glycine max* Wm82.a2.v1 reference genome retrieved from Phytozome (https://phytozome-next.jgi.doe.gov, accessed on 10 July 2023). The SNP and INDEL genetic marker numberings of 37,740,744 were obtained. Subsequently, SNP markers were screened using GATK4, with filtering parameters set as follows: FS > 60.0, DP < 4.0, QD < 2.0, QUAL < 30.0, and ReadPosRankSum < −8.0, resulting in 31,991,896 SNPs. Further filtering was performed using Plink (version 1.90) with the following command settings: −maf 0.05, −geno 0.2, and −mind 0.2, resulting in 8,256,807 SNPs [62].

### 4.3. Phenotype Acquisition

**Figure 6:**
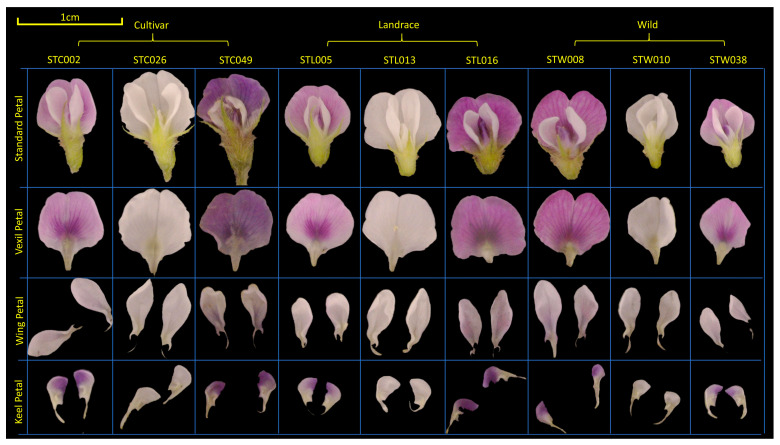
Material selection and organ decomposition of soybean flowers. The population was divided into cultivar, landrace, and wild. The flower standard petal was dissected into the vexil petal, wing petal, and keel petal. A 1 cm reference line was marked in the upper left corner in yellow.

In the present study, soybean flowers were meticulously disassembled post-extraction. Subsequently, the dissected parts were categorized into a vexil petal ×1, a wing petal ×2, and a keel petal ×2 for individual imaging (refer to Figure 6) [63]. Observation and photography of soybean flowers were conducted utilizing a three-eye microscope (ZS7050, LANGQI, Beijing, China) boasting a resolution of 48 MP. Petals were captured alongside a white reference point measuring 1 cm in diameter. The imaging setup included DEEP studio and DEEP light (SQ054, DEEP, Hangzhou, China). Seed phenotypes were assessed using a specialized machine (SC-G, Wseen, Hangzhou, China), with measurements encompassing seed length, width, average area, and average perimeter. Each variety underwent three measurements, and the results were averaged for analysis.

### 4.4. Data Extraction Based on Computer Vision

**Figure 7:**
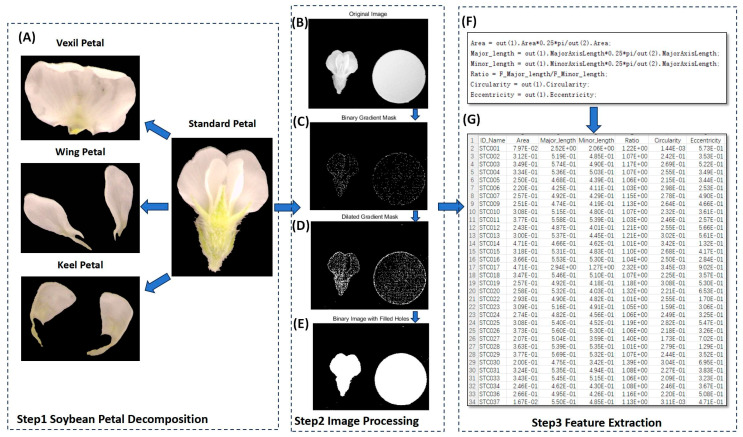
Using computer vision to extract petal phenotype workflow. (**A**) Examples of vexil petals, wing petals, and keel petals after disassembly of standard flowers. (**B**) Original image. (**C**) Binary gradient mask processing. (**D**) Dilated gradient mask processing. (**E**) Binary image with filled holes processing. (**F**) Data processing code. (**G**) Result data example.

**Figure 8:**
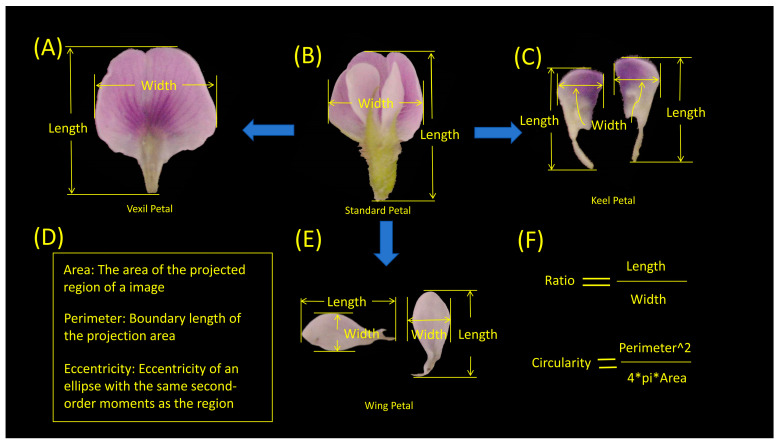
Specific quantitative indicators of the morphological phenotypes of soybean flowers. (**A**) Vexil petal length and width example. (**B**) Standard petal length and width example. (**C**) Keel petal length and width example. (**D**) Area, perimeter, and eccentricity explanation. (**E**) Wing petal length and width example. (**F**) Ratio and circularity calculation description.

In the present study, MATLAB (V 2022) was utilized to process petal images, as illustrated in Figure 7. The process entailed several key steps. (1) Binary Gradient Mask: The object to be segmented exhibited significant contrast differences from the background image. Operators calculating image gradients can detect changes in contrast. A binary mask containing the segmented soybean flower was generated by computing the gradient image and applying a threshold. The edge function and Sobel operator (in MATLAB V 2022) were utilized to determine the threshold value. Fine tuning of the threshold value and subsequent application of the edge function produced a binary mask containing segmented cells. (2) Dilated Gradient Mask: The binary gradient mask displayed high-contrast lines in the image, yet these lines did not precisely delineate the object’s outline. Gaps were present in the lines surrounding the object within the gradient mask when compared to the original image. These linear gaps could be filled by dilating the Sobel image using linear structuring elements. Two perpendicular linear structuring elements were created using the strel function in MATLAB. The binary gradient mask was dilated sequentially using the vertical and horizontal structuring elements. The imdilate function (in MATLAB) was employed for image dilation. (3) Binary Image with Filled Holes: While the dilated gradient mask provided a clear outline of the flower, there were still interior holes within the cell. These holes were filled using the imfill function in MATLAB [64]. Six phenotypes (length, width, area, perimeter, eccentricity, ratio, circularity) were extracted from the processed images of each petal type (Figure 8). A total of 24 petal phenotypic datasets, including standard (Figure 8B), vexil (Figure 8A), wing (Figure 8E), and keel (Figure 8C) petals, were obtained.

### 4.5. Analysis of Relationship

In the present study, Bioladder (https://www.bioladder.cn/web/#/chart/3, accessed on 10 September 2023) served as the platform for conducting the Pearson correlation analysis on petal and seed phenotypes, thereby calculating Pearson pairwise correlation coefficients for all traits. To validate the results, the R package stats (Version 4.3.2) was utilized for computing correlation coefficients and p values. Subsequently, the R package ggpubr (Version 0.60) facilitated the visualization of pairwise correlation scatter plots for all traits. Phenotypes exhibiting high Pearson correlation coefficients were categorized into petal and seed phenotypes. Canonical correlation analysis was then conducted on these two sets of phenotypes using the R package stats (Version 4.3.2) to determine their correlation coefficients [65]. Additionally, R (Version 4.2.1) was employed to segment each petal and seed phenotype into two intervals based on their mean values. These two groups of phenotypes were paired to investigate the presence of significant size correspondence.

### 4.6. Genome-Wide Association Studies

A total of 8,256,807 polymorphic SNP markers and 170 samples of petal phenotype and seed phenotype data were used in the present study. GWASs were performed using the R package rmvp (V 1.0.8) (parameter:priority = ”memory”, method = “MLM”) using mixed linear models (MLMs) for analysis [66,67]. False positives in the GWAS were addressed through the Bonferroni test for multiple testing corrections. Specifically, the Bonferroni correction was applied at a significance level of 1% (0.01 divided by the number of independent SNPs). The corrected significance threshold was established using −log10(1/n), where n represents the number of independent SNPs. Visualization of the GWAS results was conducted using the R package CMplot (Version 4.4.1).

### 4.7. Candidate Gene Identification

Initial peak detection was conducted for each trait based on marker positions, and candidate genes situated within the 500kb genomic region (250 kb upstream and 250 kb downstream) of each significant SNP peak were subsequently identified using the soybean reference genome *Glycine max* Wm82.a2.v1 (https://phytozome-next.jgi.doe.gov, accessed on 10 July 2023) [32]. Gene function annotation and Gene Ontology (GO) analysis were performed on the identified candidate genes utilizing resources from Soybase (https://www.soybase.org, accessed on 15 September 2023) and NCBI (https://david.ncifcrf.gov/, accessed on 10 January 2024).

### 4.8. Haplotype Analysis and Screening

According to the reference genome’s GFF3 information for *Glycine max* Wm82.a2.v1, vcftools (Version 0.1.16) was employed to extract the SNP data in VCF format files within the candidate gene segments. Subsequently, a high-throughput gene haplotype and phenotypic significance visualization script was developed using R. The R package vcfR (Version 1.15.0) was utilized for haplotype analysis on the VCF format files. Haplotypes were determined as advantageous if the number of samples with a single haplotype exceeded 5% of the total population [68]. Analysis of haplotypes and phenotypes of candidate genes, along with significant visualization, was achieved using the R packages ggpubr (Version 0.60), ggsci (Version 2.9), and ggplot2 (Version 3.4.1). This facilitated the screening of haplotypes exhibiting significant phenotypic differences and subsequent candidate gene selection.

### 4.9. Genetic Analysis of Gene Population

SNP data were used to analyze the genetic diversity of candidate genes in soybeans. The SNP data underwent filtering using plink (Version 1.9) with the following parameters: −mind 0.2, −geno 0.2, and −maf 0.05 [62]. Soybean accessions were then categorized into three populations: cultivar, landrace, and wild. Nucleotide diversity (π), the fixation index (FST), and Tajima’s D values were computed using VCFtools (Version 0.1.16) with a 20 kb sliding window (FST values calculated with a 2 kb step) [69]. Heterozygosity was determined using the -het option in vcftools (Version 0.1.16). The nucleic acid sequence of the candidate gene was subjected to BLAST analysis in NCBI (https://david.ncifcrf.gov/ (accessed on 5 July 2024)), and a phylogenetic tree was constructed based on the resultant sequences. Linkage disequilibrium analysis of the coding sequence (CDS) region within the candidate gene was conducted using Haploview (Version 4.2) [70].

### 4.10. SNP Interaction Analysis and Transcriptome Analysis

A total of 936 SNP loci were obtained by combining the SNP results of VMAL, VMIL, wing minor length, keel area, and seed phenotype obtained by the GWAS. The genotype data for each locus were coded as follows: homozygous for the reference allele as 0, homozygous for the variant allele as 2, and heterozygous as 1. The SNP data were structured as a 936 × 936 contingency table for conducting an independent sample chi-square test [71]. The formula for calculating the chi-square value for an R × R contingency table is as follows:

$$T=\frac{R-1}{R}\sum_{i-1}^{R}\frac{{\left(ni-mi\right)}^{2}}{ni+mi-2Aii}$$

Cytoscape (Version 3.7.2) was employed to create the interaction diagram. Transcriptome analysis utilized RNA-seq data from Yanjie Yao et al. to acquire three replicates each of large-grain cultivated soybeans (E1) and small-grain wild soybeans (E2) at days after flowering (DAF) 20 and DAF30, respectively [72]. Using *Glycine max* Wm82.a2.v1 as the reference genome, the data were aligned using HISAT2 (Version 2.2.1) [73]. The resulting data were then normalized, and the Fragments Per Kilobase of the exon model per million mapped fragments (FPKM) value was calculated using the following formula:

$$FPKM=\frac{ExonMappedFragments\times 10^{9}}{TotalMappedFragments\times ExonLength}$$

The expression of candidate genes was plotted to observe the expression changes in different periods [73].

## Figures and Tables

**Table 1:** Candidate gene significance. Yes represents that haplotypes are significant and No represents that haplotypes are not significant.

Phenotype	Gene ID	Gene Segment Haplotype Significance	CDS Segment Haplotype Significance
Vexil major length	*Glyma.07G231600*	Yes	Yes
	*Glyma.07G231800*	Yes	Yes
	*Glyma.07G231900*	Yes	No
	*Glyma.07G232000*	Yes	Yes
	*Glyma.07G232300*	Yes	Yes
	*Glyma.07G232400*	Yes	Yes
	*Glyma.07G232500*	Yes	Yes
	*Glyma.07G233300*	Yes	Yes
	*Glyma.07G233400*	Yes	Yes
	*Glyma.07G233500*	Yes	No
	*Glyma.07G233900*	Yes	Yes
	*Glyma.07G234000*	Yes	Yes
	*Glyma.07G234100*	Yes	Yes
	*Glyma.07G234200*	Yes	Yes
	*Glyma.07G234500*	Yes	Yes
	*Glyma.07G234800*	Yes	Yes
	*Glyma.07G234900*	Yes	Yes
	*Glyma.07G235000*	Yes	Yes
	*Glyma.07G235200*	Yes	No
	*Glyma.07G235400*	Yes	No
	*Glyma.07G235500*	Yes	Yes
	*Glyma.07G234300*	Yes	Yes
Vexil minor length	*Glyma.07G231600*	Yes	Yes
	*Glyma.07G231800*	Yes	Yes
	*Glyma.07G231900*	Yes	No
	*Glyma.07G232000*	Yes	Yes
	*Glyma.07G232200*	Yes	Yes
	*Glyma.07G232300*	Yes	Yes
	*Glyma.07G232400*	Yes	Yes
	*Glyma.07G232500*	Yes	Yes
	*Glyma.07G232800*	Yes	Yes
	*Glyma.07G233300*	Yes	Yes
	*Glyma.07G233400*	Yes	Yes
	*Glyma.07G233600*	Yes	Yes
	*Glyma.07G234200*	Yes	Yes
	*Glyma.07G234500*	Yes	Yes
	*Glyma.07G234800*	Yes	Yes
	*Glyma.07G234900*	Yes	Yes
	*Glyma.07G235000*	Yes	Yes
	*Glyma.07G235200*	Yes	No
	*Glyma.07G235400*	Yes	No
	*Glyma.07G235500*	Yes	Yes
Wing minor length	*Glyma.18G043700*	Yes	Yes
	*Glyma.18G045700*	Yes	Yes
	*Glyma.18G046000*	Yes	No
	*Glyma.18G047900*	Yes	No
	*Glyma.18G276800*	Yes	Yes
	*Glyma.18G277400*	Yes	Yes
	*Glyma.18G278600*	Yes	No
	*Glyma.18G277800*	Yes	Yes
	*Glyma.18G278900*	Yes	Yes
	*Glyma.18G280700*	Yes	Yes

## Data Availability

The author confirms that all necessary data, including analysis data, to verify the article’s contents are provided alongside the article on https://pan.baidu.com/s/1W3mKOCNUwyqrsSv7tqgbtA?pwd=30lt (accessed on 5 July 2024).

## Supplementary Materials

The following supporting information can be downloaded at https://www.mdpi.com/article/10.3390/ijms25147622/s1.

## References

[1] Li Y.H., Reif J.C., Hong H.L., Li H.H., Liu Z.X., Ma Y.S., Li J., Tian Y., Li Y.F., Li W.B. (2018). Genome-wide association mapping of QTL underlying seed oil and protein contents of a diverse panel of soybean accessions. Plant Sci..

[2] Hymowitz T., Collins F.I., Panczner J., Walker W.M. (1972). Relationship Between the Content of Oil, Protein, and Sugar in Soybean Seed. Agron. J..

[3] Jiang G.L., Chen P.Y., Zhang J.P., Florez-Palacios L., Zeng A.L., Wang X.Z., Bowen R.A., Miller A., Berry H. (2018). Genetic Analysis of Sugar Composition and Its Relationship with Protein, Oil, and Fiber in Soybean. Crop Sci..

[4] Graham P.H., Vance C.P. (2003). Legumes: Importance and constraints to greater use. Plant Physiol..

[5] Johnson K., Lenhard M. (2011). Genetic control of plant organ growth. New Phytol..

[6] Sussex T.A., Ian M. (1972). Patterns in Plant Development.

[7] Feng G.P., Qin Z.X., Yan J.Z., Zhang X.R., Hu Y.X. (2011). Arabidopsis *ORGAN SIZE RELATED1* regulates organ growth and final organ size in orchestration with *ARGOS* and *ARL*. New Phytol..

[8] Li S.J., Liu Y.J., Zheng L.Y., Chen L.L., Li N., Corke F., Lu Y.R., Fu X.D., Zhu Z.G., Bevan M.W. (2012). The plant-specific G protein? subunit AGG3 influences organ size and shape in Arabidopsis thaliana. New Phytol..

[9] Dafni A., Kevan P.G. (1997). Flower size and shape: Implications in pollination. Isr. J. Plant Sci..

[10] Shpak E.D., Berthiaume C.T., Hill E.J., Torii K.U. (2004). Synergistic interaction of three ERECTA-family receptor-like kinases controls *Arabidopsis* organ growth and flower development by promoting cell proliferation. Development.

[11] Song P., Wang J.L., Guo X.Y., Yang W.N., Zhao C.J. (2021). High-throughput phenotyping: Breaking through the bottleneck in future crop breeding. Crop J..

[12] Patrício D.I., Rieder R. (2018). Computer vision and artificial intelligence in precision agriculture for grain crops: A systematic review. Comput. Electron. Agric..

[13] Affortit P., Effa-Effa B., Ndoye M.S., Moukouanga D., Luchaire N., Cabrera-Bosquet L., Perálvarez M., Pilloni R., Welcker C., Champion A. (2022). Physiological and genetic control of transpiration efficiency in African rice, *Oryza glaberrima* Steud. J. Exp. Bot..

[14] Guo S.J., Zhou G.L., Wang J.L., Lu X.J., Zhao H., Zhang M.G., Guo X.Y., Zhang Y. (2022). High-Throughput Phenotyping Accelerates the Dissection of the Phenotypic Variation and Genetic Architecture of Shank Vascular Bundles in Maize (*Zea mays* L.). Plants.

[15] Nagle M.F., Yuan J.L., Kaur D., Ma C., Peremyslova E., Jiang Y., de Rivera A.N., Jawdy S., Chen J.G., Feng K. (2024). GWAS supported by computer vision identifies large numbers of candidate regulators of in planta regeneration in Populus trichocarpa. G3-Genes Genomes Genet..

[16] Mizukami Y., Fischer R.L. (2000). Plant organ size control: AINTEGUMENTA regulates growth and cell numbers during organogenesis. Proc. Natl. Acad. Sci. USA.

[17] Zhou S., Yang T., Mao Y., Liu Y., Chen J. (2021). The F-box protein MIO1/SLB1 regulates organ size and leaf movement in Medicago truncatula. J. Exp. Bot..

[18] Dehghan A. (2018). Genome-Wide Association Studies. Methods Mol. Biol..

[19] Thomson M.J. (2014). High-Throughput SNP Genotyping to Accelerate Crop Improvement. Plant Breed. Biotechnol..

[20] Korte A., Farlow A. (2013). The advantages and limitations of trait analysis with GWAS: A review. Plant Methods.

[21] Ibrahim A.K., Zhang L.W., Niyitanga S., Afzal M.Z., Xu Y., Zhang L.L., Zhang L.M., Qi J.M. (2020). Principles and approaches of association mapping in plant breeding. Trop. Plant Biol..

[22] Tian F., Bradbury P.J., Brown P.J., Hung H., Sun Q., Flint-Garcia S., Rocheford T.R., McMullen M.D., Holland J.B., Buckler E.S. (2011). Genome-wide association study of leaf architecture in the maize nested association mapping population. Nat. Genet..

[23] Huang X.H., Wei X.H., Sang T., Zhao Q.A., Feng Q., Zhao Y., Li C.Y., Zhu C.R., Lu T.T., Zhang Z.W. (2010). Genome-wide association studies of 14 agronomic traits in rice landraces. Nat. Genet..

[24] Zhang J.P., Song Q.J., Cregan P.B., Nelson R.L., Wang X.Z., Wu J.X., Jiang G.L. (2015). Genome-wide association study for flowering time, maturity dates and plant height in early maturing soybean (*Glycine max*) germplasm. BMC Genom..

[25] Lin F., Wani S.H., Collins P.J., Wen Z.X., Li W.L., Zhang N., McCoy A.G., Bi Y.D., Tan R.J., Zhang S.C. (2020). QTL mapping and GWAS for identification of loci conferring partial resistance to *Pythium sylvaticum* in soybean (*Glycine max* (L.) Merr). Mol. Breed..

[26] Zhang J.P., Song Q.J., Cregan P.B., Jiang G.L. (2016). Genome-wide association study, genomic prediction and marker-assisted selection for seed weight in soybean (*Glycine max*). Theor. Appl. Genet..

[27] Wu C.J., Mozzoni L.A., Moseley D., Hummer W., Ye H., Chen P.Y., Shannon G., Nguyen H. (2019). Genome-wide association mapping of flooding tolerance in soybean. Mol. Breed..

[28] Li D.M., Zhao X., Han Y.P., Li W.B., Xie F.T. (2019). Genome-wide association mapping for seed protein and oil contents using a large panel of soybean accessions. Genomics.

[29] Do T.D., Vuong T.D., Dunn D., Clubb M., Valliyodan B., Patil G., Chen P.Y., Xu D., Nguyen H.T., Shannon J.G. (2019). Identification of new loci for salt tolerance in soybean by high-resolution genome-wide association mapping. BMC Genom..

[30] Du W.K., Ning L.H., Liu Y.S., Zhang S.X., Yang Y.M., Wang Q., Chao S.Q., Yang H., Huang F., Cheng H. (2020). Identification of loci and candidate gene *GmSPX-RING1* responsible for phosphorus efficiency in soybean via genome-wide association analysis. BMC Genom..

[31] Chang F.G., Lv W.H., Lv P.Y., Xiao Y.T., Yan W.L., Chen S., Zheng L.Y., Xie P., Wang L., Karikari B. (2021). Exploring genetic architecture for pod-related traits in soybean using image-based phenotyping. Mol. Breed..

[32] Hao D., Cheng H., Yin Z., Cui S., Zhang D., Yu W.D. (2012). Identification of single nucleotide polymorphisms and haplotypes associated with yield and yield components in soybean (*Glycine max*) landraces across multiple environments. TAG Theor. Appl. Genet..

[33] Matsuda H., Nakayasu M., Aoki Y., Yamazaki S., Nagano A.J., Yazaki K., Sugiyama A. (2020). Diurnal metabolic regulation of isoflavones and soyasaponins in soybean roots. Plant Direct.

[34] Bobo E., Munosiyei P., Jinga P., Zingoni E. (2019). Functional Characterisation of a Calmodulin-Binding Receptor-Like Cytoplasmic Kinase (GmCBRLCK1) in *Glycine max* (L.) Merr. using Bioinformatic Tools. Int. Ann. Sci..

[35] Du J., Wang S.D., He C.M., Zhou B., Ruan Y.L., Shou H.X. (2017). Identification of regulatory networks and hub genes controlling soybean seed set and size using RNA sequencing analysis. J. Exp. Bot..

[36] Chen Q., Liu B.Q., Ai L.J., Yan L., Lin J., Shi X.L., Zhao H.T., Wei Y., Feng Y., Liu C.J. (2022). QTL and candidate genes for heterophylly in soybean based on two populations of recombinant inbred lines. Front. Plant Sci..

[37] Van den Oord E., Neale B. (2004). Will haplotype maps be useful for finding genes?. Mol. Psychiatry.

[38] Poland J.A., Bradbury P.J., Buckler E.S., Nelson R.J. (2011). Genome-wide nested association mapping of quantitative resistance to northern leaf blight in maize. Proc. Natl. Acad. Sci. USA.

[39] Bheemanahalli R., Wang C.X., Bashir E., Chiluwal A., Pokharel M., Perumal R., Moghimi N., Ostmeyer T., Caragea D., Jagadish S.V.K. (2021). Classical phenotyping and deep learning concur on genetic control of stomatal density and area in sorghum. Plant Physiol..

[40] Hu Z.B., Zhang H.R., Kan G.Z., Ma D.Y., Zhang D., Shi G.X., Hong D.L., Zhang G.Z., Yu D.Y. (2013). Determination of the genetic architecture of seed size and shape via linkage and association analysis in soybean (*Glycine max* L. Merr.). Genetica.

[41] Mansur L.M., Lark K.G., Kross H., Oliveira A. (1993). Interval mapping of quantitative trait loci for reproductive, morphological, and seed traits of soybean (*Glycine max* L.). Theor. Appl. Genet..

[42] Wang D., Graef G.L., Procopiuk A.M., Diers B.W. (2004). Identification of putative QTL that underlie yield in interspecific soybean backcross populations. Theor. Appl. Genet..

[43] Gonzalez N., Vanhaeren H., Inzé D. (2012). Leaf size control: Complex coordination of cell division and expansion. Trends Plant Sci..

[44] Klein J., Saedler H., Huijser P. (1996). A new family of DNA binding proteins includes putative transcriptional regulators of the Antirrhinum majus floral meristem identity gene SQUAMOSA. Mol. Gen. Genet..

[45] Becraft P.W., Bongard-Pierce D.K., Sylvester A.W., Poethig R.S., Freeling M. (1990). The liguleless-1 gene acts tissue specifically in maize leaf development. Dev. Biol..

[46] Cardon G.H., Hohmann S., Nettesheim K., Saedler H., Huijser P. (1997). Functional analysis of the Arabidopsis thaliana SBP-box gene SPL3: A novel gene involved in the floral transition. Plant J. Cell Mol. Biol..

[47] Cardon G., Höhmann S., Klein J., Nettesheim K., Saedler H., Huijser P. (1999). Molecular characterisation of the Arabidopsis SBP-box genes. Gene.

[48] Lännenpää M., Jänönen I., Hölttä-Vuori M., Gardemeister M.M., Sopanen T. (2010). A new SBP-box gene BpSPL1 in silver birch (Betula pendula). Physiol. Plant..

[49] Schmid M., Uhlenhaut N.H., Godard F., Demar M., Bressan R., Weigel D., Lohmann J.U. (2003). Dissection of floral induction pathways using global expression analysis. Development.

[50] Unte U.S., Sorensen A.M., Pesaresi P., Gandikota M., Leister D., Saedler H., Huijser P. (2003). *SPL8*, an SBP-Box gene that affects pollen sac development in Arabidopsis. Plant Cell.

[51] Breuninger H., Lenhard M. (2010). Control of Tissue and Organ Growth in Plants. Curr. Top. Dev. Biol..

[52] Mizukami Y. (2001). A matter of size: Developmental control of organ size in plants. Curr. Opin. Plant Biol..

[53] Tombuloglu G., Aldahnem A., Tombuloglu H., Slimani Y., Akhtar S., Hakeem K.R., Almessiere M.A., Baykal A., Ercan I., Manikandan A. (2024). Uptake and bioaccumulation of iron oxide nanoparticles (Fe_3_O_4_) in barley (*Hordeum vulgare* L.): Effect of particle-size. Environ. Sci. Pollut. Res..

[54] Went F.W. (2003). The Effect of Temperature on Plant Growth. Annu. Rev. Plant Physiol..

[55] Powell A.E., Lenhard M. (2012). Control of Organ Size in Plants. Curr. Biol..

[56] Barrow N.J., Hartemink A.E.E. (2023). The effects of pH on nutrient availability depend on both soils and plants. Plant Soil.

[57] Scavo C.M. (2019). Giovanni, Plant allelochemicals: Agronomic, nutritional and ecological relevance in the soil system. Plant Soil.

[58] Wang X., Li Y., Wang X., Li X., Dong S. (2022). Physiology and metabonomics reveal differences in drought resistance among soybean varieties. Bot. Stud..

[59] Rairdin A., Fotouhi F., Zhang J.P., Mueller D.S., Ganapathysubramanian B., Singh A.K., Dutta S., Sarkar S., Singh A. (2022). Deep learning-based phenotyping for genome wide association studies of sudden death syndrome in soybean. Front. Plant Sci..

[60] Zhong H.B. (2015). Comparative Analysis of Exon Sequencing Data between BGISEQ-500, Hiseq 4000 and Hiseq X Ten Sequencing Platforms.

[61] Van der Auwera G.A. (2017). Somatic variation discovery with GATK4. Cancer Res..

[62] Purcell S., Neale B., Todd-Brown K., Thomas L., Ferreira M.A.R., Bender D., Maller J., Sklar P., de Bakker P.I.W., Daly M.J. (2007). PLINK: A tool set for whole-genome association and population-based linkage analyses. Am. J. Hum. Genet..

[63] Chen L.Y., Nan H.Y., Kong L.P., Yue L., Yang H., Zhao Q.S., Fang C., Li H.Y., Cheng Q., Lu S.J. (2020). Soybean *AP1* homologs control flowering time and plant height. J. Integr. Plant Biol..

[64] Zhang Z. (2013). Proficient in Matlab Digital Image Processing and Recognition.

[65] Hotelling H. (1935). Relations Between Two Sets of Variates. Biometrika.

[66] Yin L.L., Zhang H.H., Tang Z.S., Xu J.Y., Yin D., Zhang Z.W., Yuan X.H., Zhu M.J., Zhao S.H., Li X.Y. (2021). rMVP: A Memory-efficient, Visualization-enhanced, and Parallel-accelerated Tool for Genome-wide Association Study. Genom. Proteom. Bioinform..

[67] Zhang Z.W., Ersoz E., Lai C.Q., Todhunter R.J., Tiwari H.K., Gore M.A., Bradbury P.J., Yu J.M., Arnett D.K., Ordovas J.M. (2010). Mixed linear model approach adapted for genome-wide association studies. Nat. Genet..

[68] Smith A.N., Barratt B.J., Stevens H., Hughes W., Gough S.C., Cordell H.J., Dudbridge F., Nutland S., Heward J., Tuomilehto J. (2013). Haplotype tagging for the identification of common disease genes. Nat. Genet..

[69] Danecek P., Auton A., Abecasis G., Albers C.A., Banks E., DePristo M.A., Handsaker R.E., Lunter G., Marth G.T., Sherry S.T. (2011). The variant call format and VCFtools. Bioinformatics.

[70] Barrett J.C. (2009). Haploview: Visualization and analysis of SNP genotype data. Cold Spring Harb. Protoc..

[71] McHugh M.L. (2013). The Chi-square test of independence. Biochem. Medica.

[72] Yao Y.J., Xiong E.R., Qu X.L., Li J.F., Liu H.L., Quan L.P., Lu W.Y., Zhu X.L., Chen M.L., Li K. (2023). WGCNA and transcriptome profiling reveal hub genes for key development stage seed size/oil content between wild and cultivated soybean. BMC Genom..

[73] Wen G. A Simple Process of RNA-Sequence Analyses by Hisat2, Htseq and DESeq2. Proceedings of the International Conference on Biomedical Engineering.

