# Supplementary figures and images for: Genetic Analysis of Soybean Flower Size Phenotypes Based on Computer Vision and Genome-Wide Association Studies
Source: Int J Mol Sci. 2024 Jul 11;25(14):7622. doi: 10.3390/ijms25147622 (PMC11277310; doi:10.3390/ijms25147622)

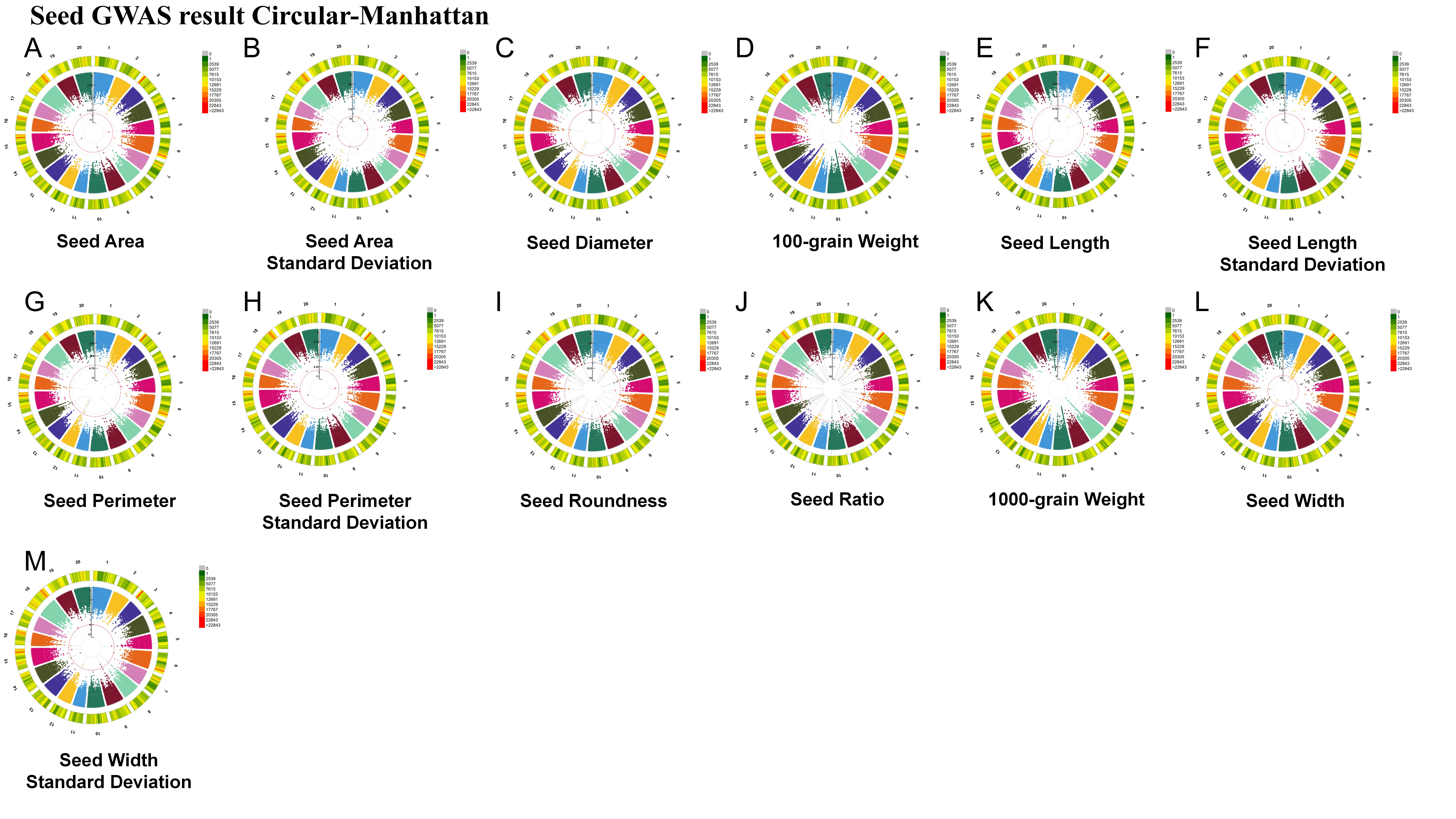

Supplement: Supplementary file 1 [file ijms-25-07622-s001.zip › Supplementary Figure/Supplementary Figure S1 Seed GWAS result Circular Manhattan.jpg]

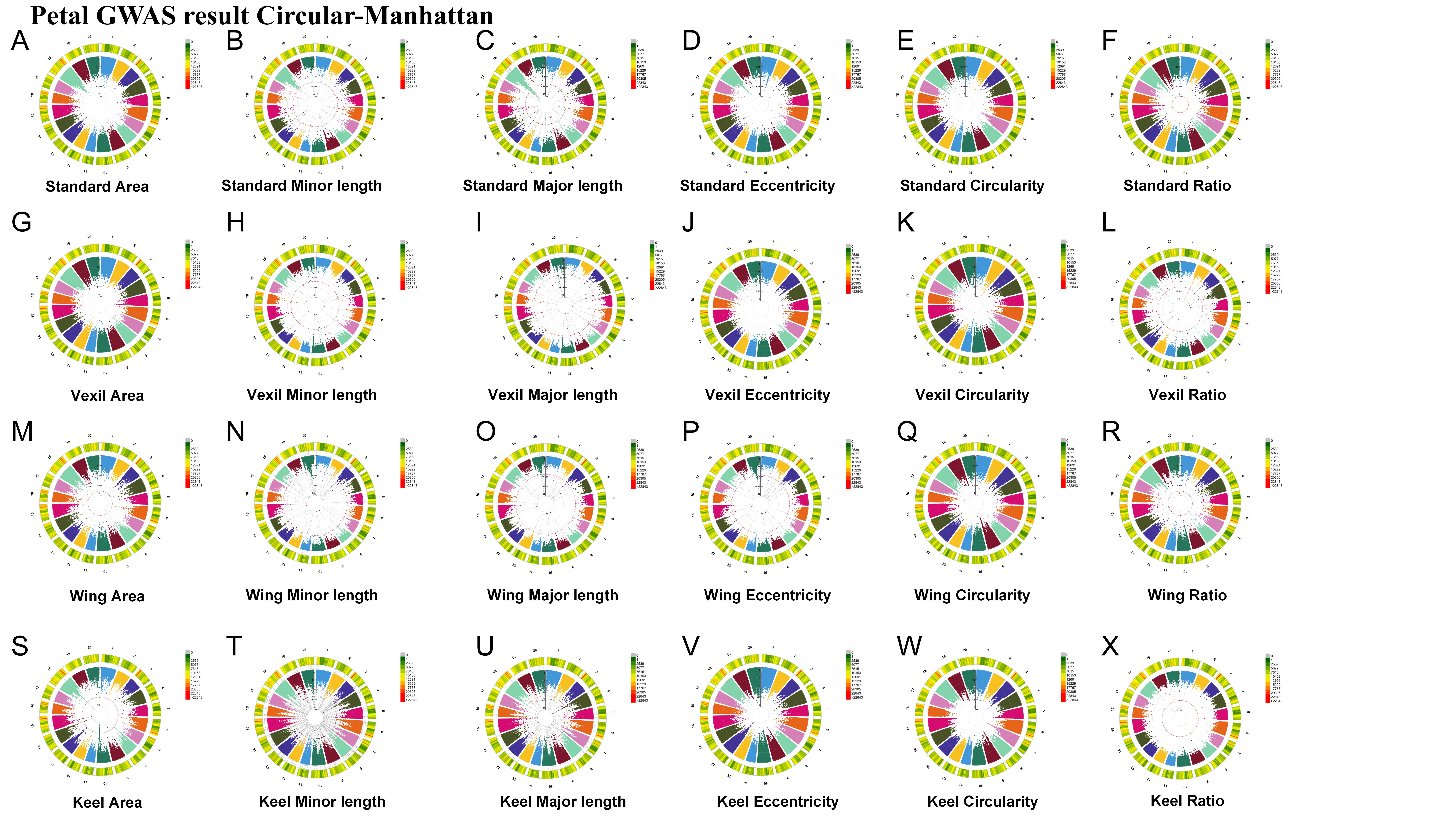

Supplement: Supplementary file 1 [file ijms-25-07622-s001.zip › Supplementary Figure/Supplementary Figure S2 Petal GWAS result Circular Manhattan.jpg]

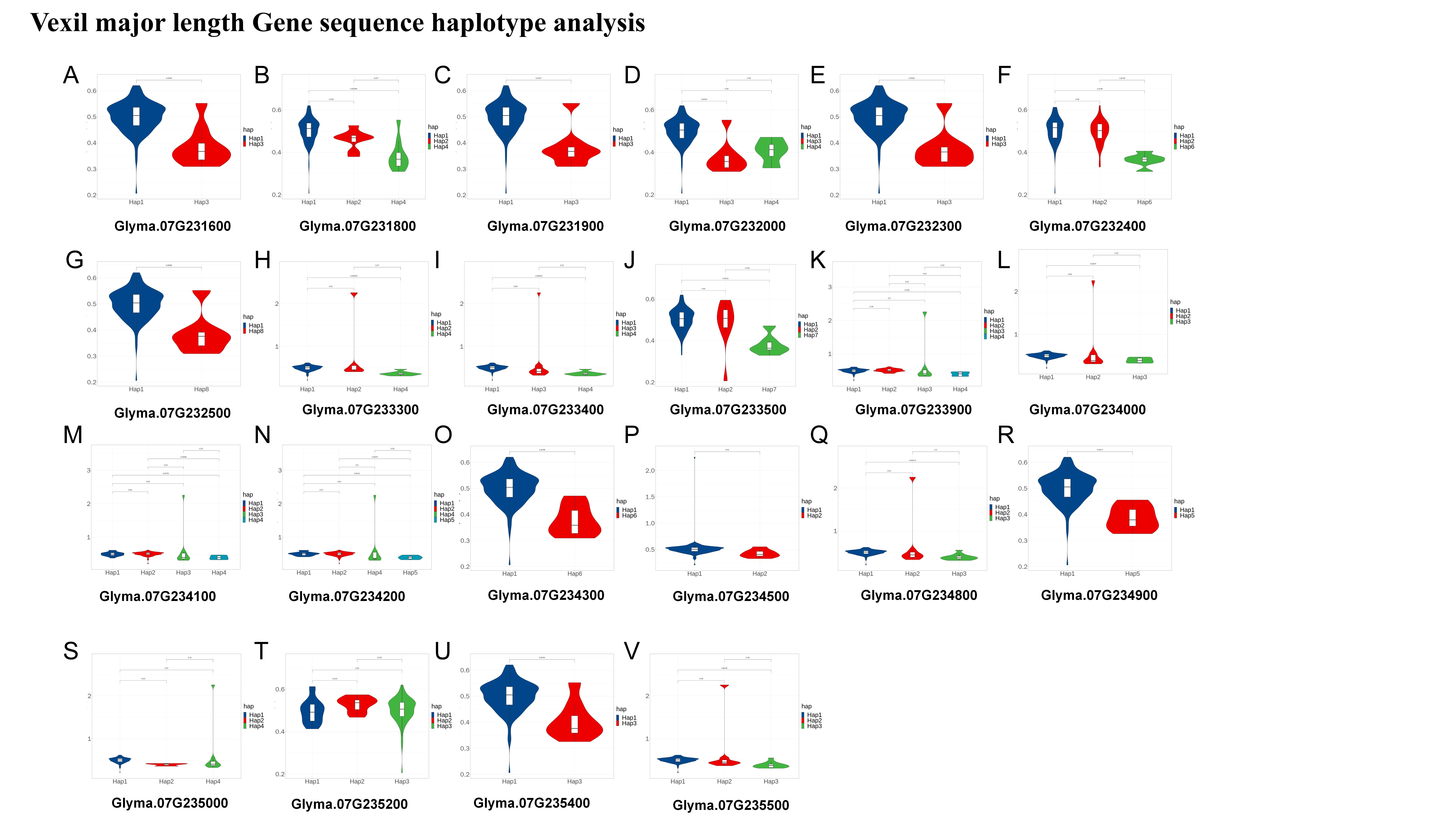

Supplement: Supplementary file 1 [file ijms-25-07622-s001.zip › Supplementary Figure/Supplementary Figure S3 Vexil major length Gene sequence haplotype analysis.jpg]

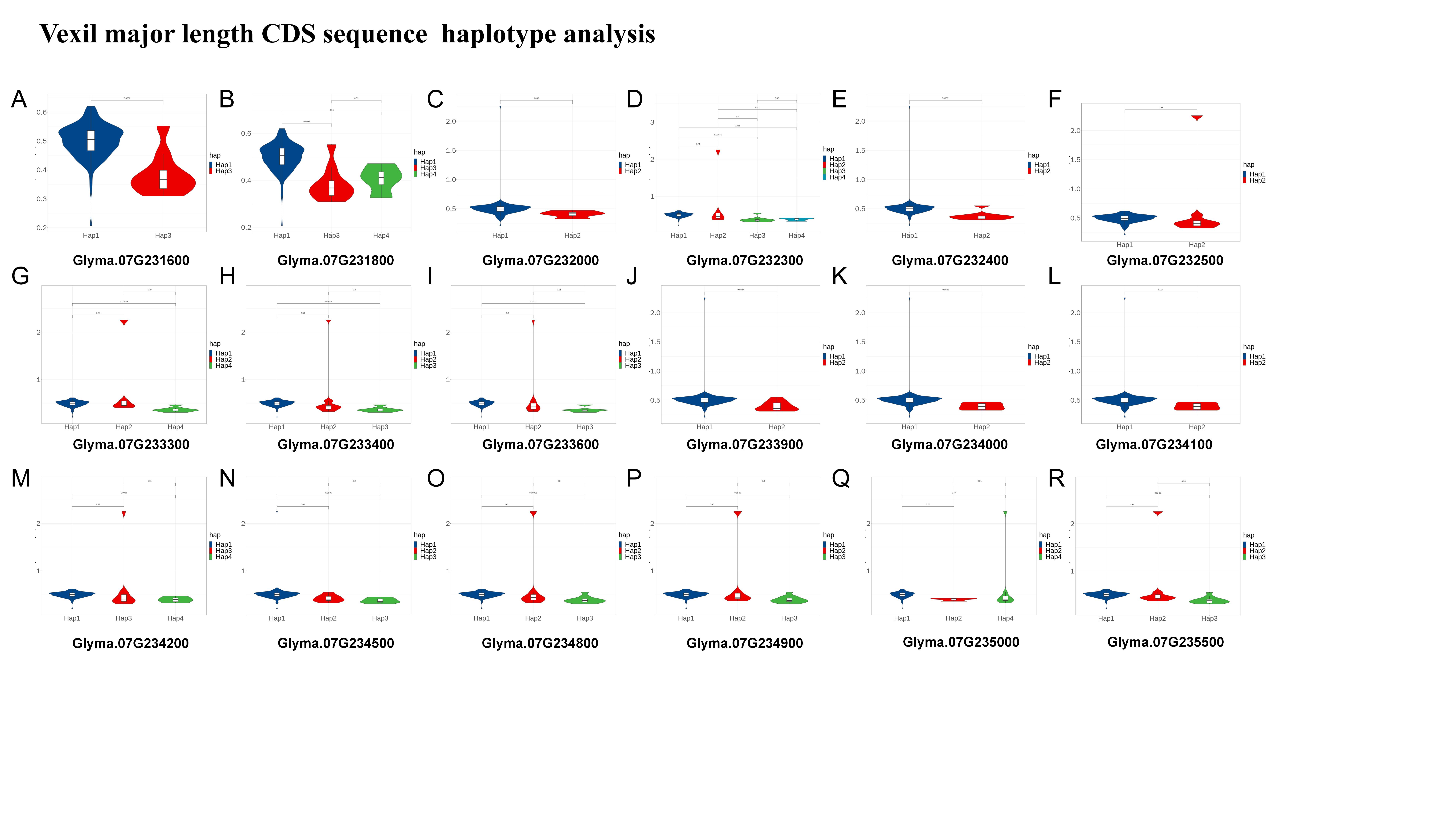

Supplement: Supplementary file 1 [file ijms-25-07622-s001.zip › Supplementary Figure/Supplementary Figure S4 Vexil major length CDS sequence haplotype analysis.jpg]

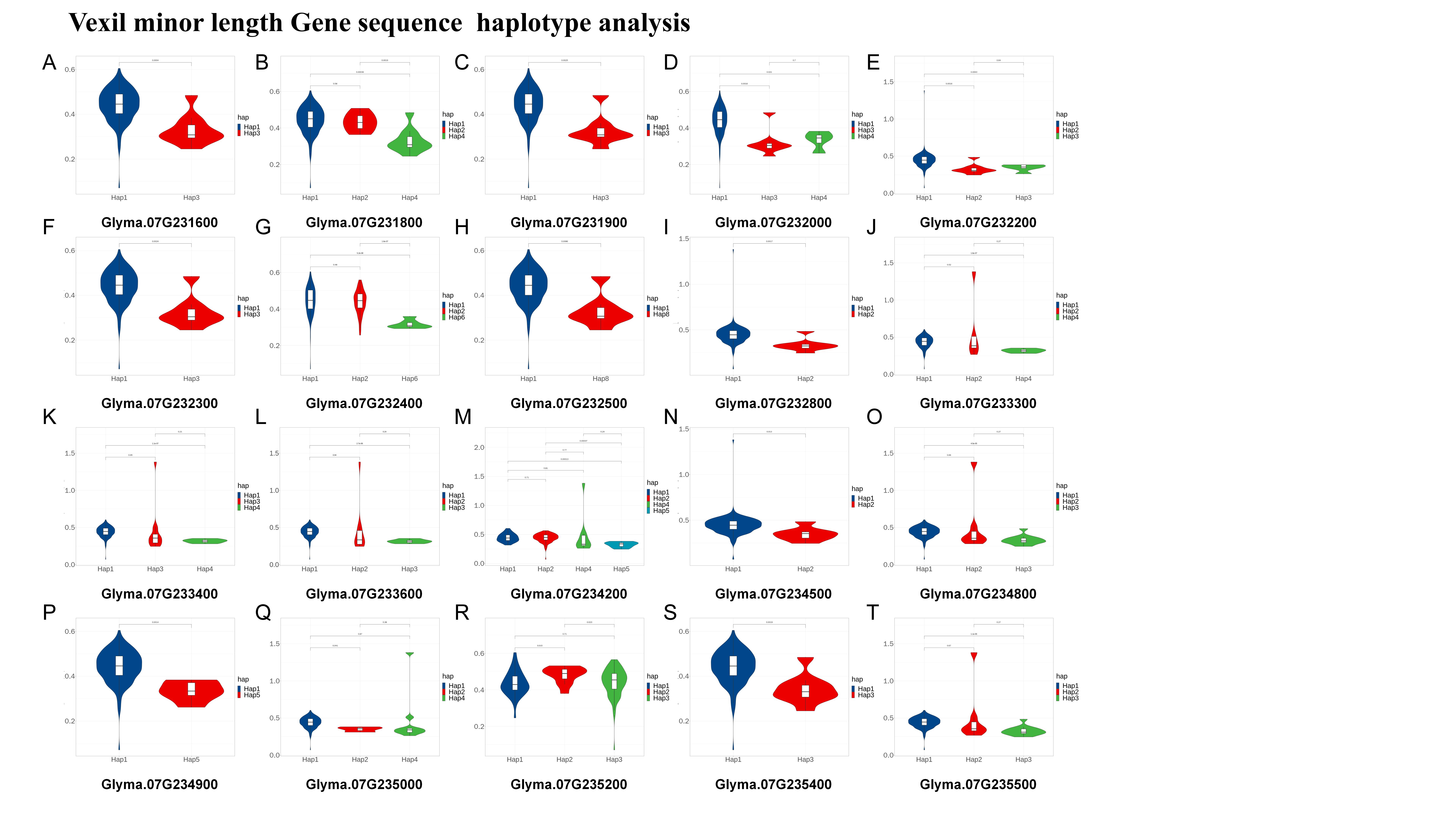

Supplement: Supplementary file 1 [file ijms-25-07622-s001.zip › Supplementary Figure/Supplementary Figure S5 Vexil minor length Gene sequence haplotype analysis.jpg]

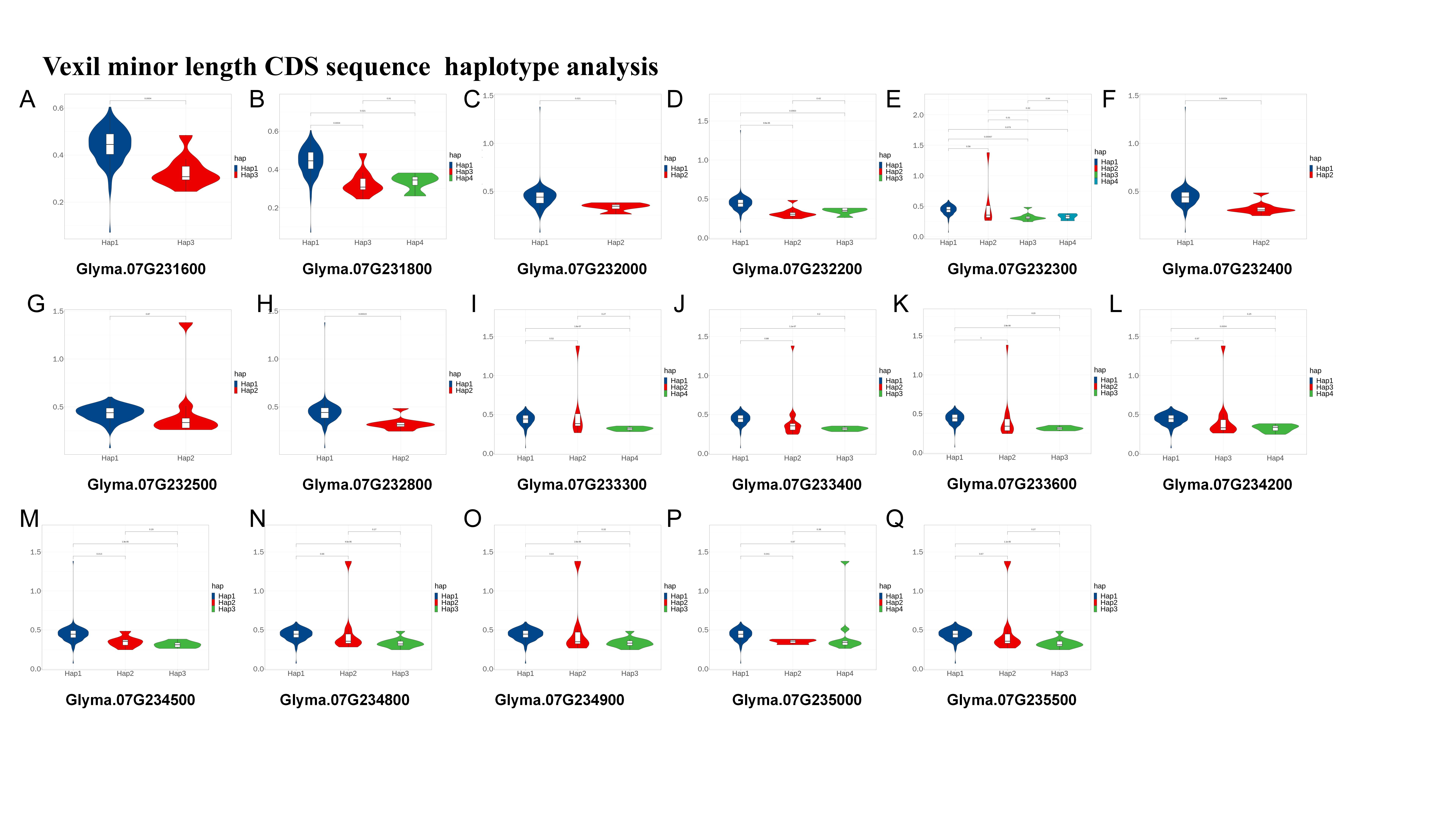

Supplement: Supplementary file 1 [file ijms-25-07622-s001.zip › Supplementary Figure/Supplementary Figure S6 Vexil minor length CDS sequence haplotype analysis.jpg]

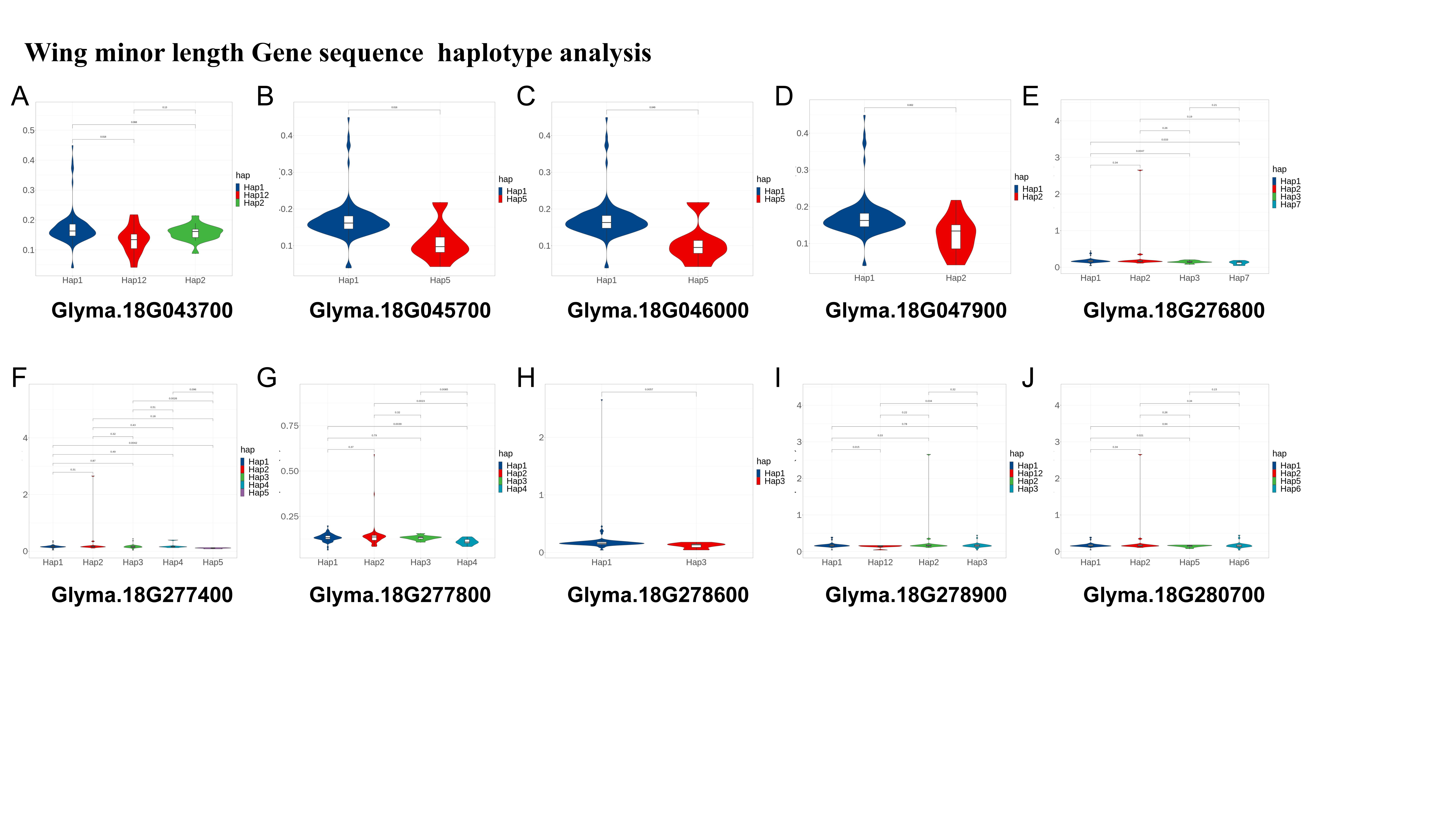

Supplement: Supplementary file 1 [file ijms-25-07622-s001.zip › Supplementary Figure/Supplementary Figure S7 Wing minor length Gene sequence haplotype analysis.jpg]

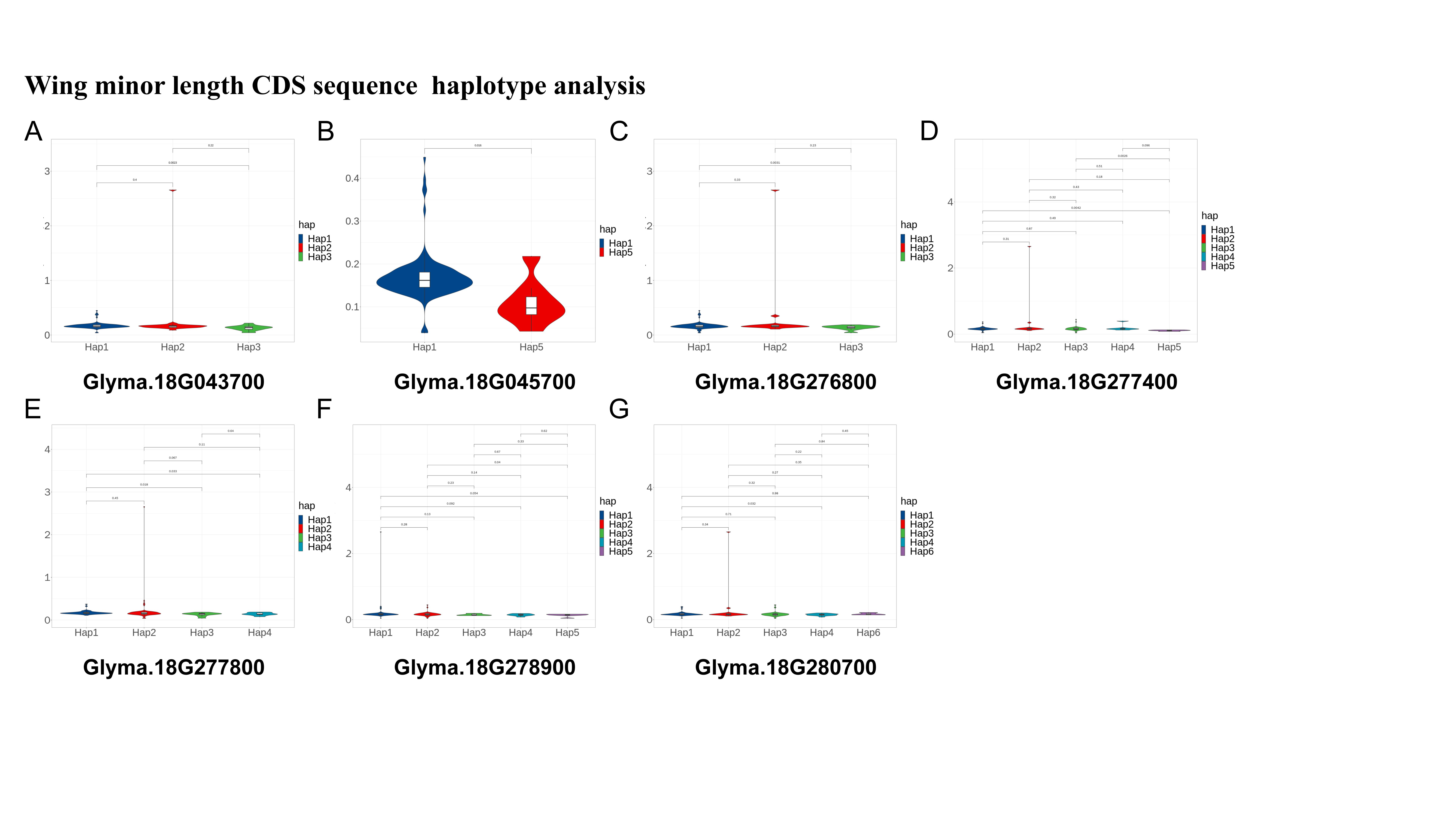

Supplement: Supplementary file 1 [file ijms-25-07622-s001.zip › Supplementary Figure/Supplementary Figure S8 Wing minor length CDS sequence haplotype analysis.jpg]

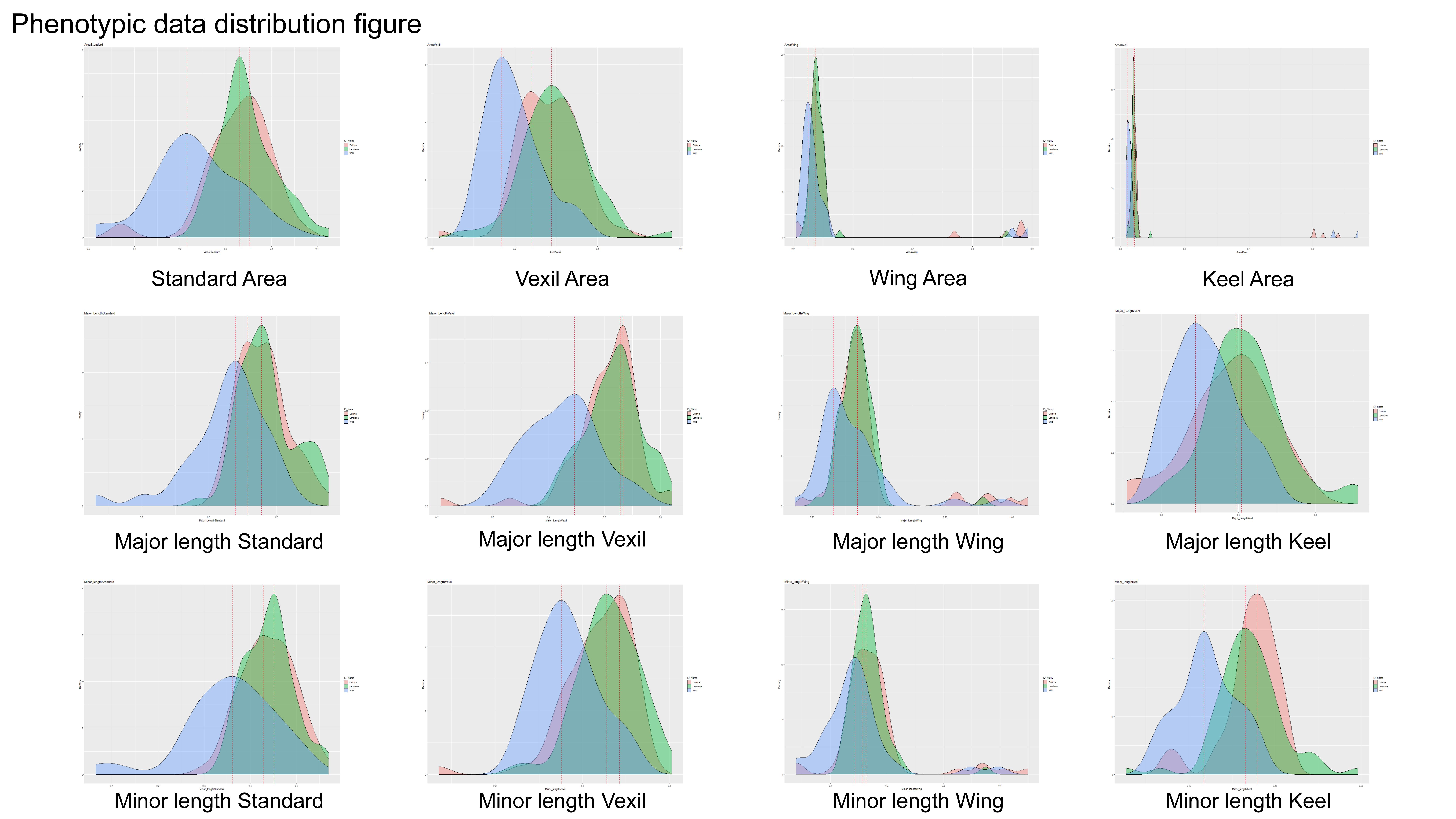

Supplement: Supplementary file 1 [file ijms-25-07622-s001.zip › Supplementary Figure/Supplementary Figure S9 phenotypic data distribution figure.jpg]
